# Calomel in Acute Articular Rheumatism

**Published:** 1849-11

**Authors:** 


*Calomel on Acute Articular Rheumatism.*—Dr. Leclercq has published in *L' Union Medicale*, several cases of acute articular rheumatism successfully treated by small doses of calomel. Dr. Law, of Dublin, had, so early as 1838, pointed out the advantages of this practice, as Dr. Trousseau, of Paris, has likewise done, in his book on therapeutics; but these physicians used to combine quinine with the calomel, and Dr. Leclercq has obtained very good results by calomel alone. These were the different steps of the treatment:

1. Bleeding, if the subject be plethoric.
2. Calomel in divided doses—viz: one grain of calomel in about a drachm of white sugar, to be divided into twelve papers; one to be taken every hour.

3. An opiate at night.
4. Cooling drinks.
5. Poultices, sprinkled with laudanum, on the painful joints.

This method has been found to counteract as well, if not better, cardiac complications. Lemon-juice, on the other hand, seems to be a greater favorite in this country, and has yielded excellent results.—*Lancet.*

